# Buffalo Academy of Medicine

**Published:** 1903-04

**Authors:** N. L. Burnham


### Buffalo Academy of Medicine.

*Section on Medicine, January 13, 1903.*

Reported by N. L. BURNHAM, M. D., Secretary.

The Medical Section of the Buffalo Academy of Medicine held its regular meeting in the Academy Rooms, Public Library Building, Tuesday evening, March 10, 1903, Dr. A. E. Woehnert in the chair.

Dr. A. L. Benedict presented a paper on

LOCAL GEOGRAPHIC CONDITIONS AND POPULATION AS BEARING ON THE WATER SUPPLY AND SEWERAGE OF BUFFALO.[1]

DISCUSSION.

Dr. Walter D. Greene, Health Commissioner of Buffalo, in opening the discussion, said that the water supply of Buffalo is not as good as it was ten years ago. There was very appreciable pollution of Buffalo's water supply during 1901—the Pan-American year. The amount of chlorine has increased from 3 parts to between 8½ and 11 parts per 1,000,000 during the past ten years. During the same period the albuminoïd ammonia has increased to three times the amount it was. He recommended a filtration plant.

Dr. Charles Cary asked Dr. Greene whether cases of typhoid fever, which develop in West Seneca and are removed to the Buffalo Hospitals, are counted as city cases? Also what

1. To be published.

is the ordinary impurity of water where no sewage is poured in? Could Smokes Creek be turned inside the breakwater?

Dr. Greene replied that the ordinary impurity of water, as nearly as he could state, was chlorine 3 per 1,000,000; albuminoid ammonia, .03 to .04 per 1,000,000. There is a canal which runs from inside the breakwater nearly to Smokes Creek, and an effort had been made to get the right to extend this canal to the creek, but as yet the steel plant authorities had not granted the request. We have twice as many cases of typhoid fever now as we had a year ago. Cases coming from Stony Point and reported from the hospitals or private residences are counted as city cases.

Dr. Henry R. Hopkins said that during the past twenty years we have had from eight to twenty times as much typhoid as any intelligent community should have. Bird Island pier is no screen for sewage. Lake Erie is becoming constantly more contaminated and we must have a filtration plant.

Dr. Chas. R. Borzilleri considered the West Seneca soil in the vicinity of the Steel Plant to be seriously contaminated with the typhoid bacillus.

Dr. George E. Fell said the Canadian channel current brings water to our intake. This is a tremendously heavy current and he did not believe Smokes Creek water ever reaches our source of water supply.

Dr. Bernard Bartow had spent the last ten summers along the American shore near Eighteen Mile Creek. There is a current of half a mile an hour toward Buffalo on a calm day. With a breeze in the same direction the current is changed to a mile an hour, while with a heavy breeze the current is increased to two miles an hour. With the present construction of the sea wall this current carries Smokes Creek water to our source of water supply.

Dr. E. H. Ballou, health officer at West Seneca, stated that during the past six months one physician had reported 100 cases of typhoid fever occurring in that town. At the present time there is being carried on a thorough house to house inspection. There soon will be a population of 50,000 people upon four square miles of land, all of which is to be sewered into Smokes Creek.

Dr. B. G. Long pointed out that the children should be supplied with boiled water at school.

Health Commissioner Greene said that the subject of boiled water in the schools was an important one, and that today Superintendent Emerson and himself had visited some of the schools endeavoring to solve this problem.

